# Risk for Facial Palsy after COVID-19 Vaccination, South Korea, 2021–2022

**DOI:** 10.3201/eid3011.240610

**Published:** 2024-11

**Authors:** Dongwon Yoon, Kyungyeon Jung, Ju Hwan Kim, Hwa Yeon Ko, Byeol-A Yoon, Ju-Young Shin, CoVaSC Investigators

**Affiliations:** Sungkyunkwan University Department of Biohealth Regulatory Science, Suwon, South Korea (D. Yoon, K. Jung, J.H. Kim, J.-Y. Shin); Sungkyunkwan University School of Pharmacy, Suwon (D. Yoon, J.H. Kim, H.Y. Ko, J.-Y. Shin); Dong-A University College of Medicine Department of Neurology, Busan, South Korea (B.-A. Yoon); Sungkyunkwan University Samsung Advanced Institute for Health Sciences & Technology (SAIHST), Seoul, South Korea (J.-Y. Shin)

**Keywords:** COVID-19, respiratory infections, severe acute respiratory syndrome coronavirus 2, SARS-CoV-2, SARS, coronavirus disease, zoonoses, viruses, coronavirus, facial palsy, vaccines, South Korea

## Abstract

We conducted a self-controlled case series study to investigate the association between COVID-19 vaccination and facial palsy (FP) in South Korea. We used a large immunization registry linked with the national health information database. We included 44,564,345 patients >18 years of age who received >1 dose of COVID-19 vaccine (BNT162b2, mRNA-1273, ChAdOx1 nCoV-19, or Ad.26.COV2.S) and had an FP diagnosis and corticosteroid prescription within 240 days postvaccination. We compared FP incidence in a risk window (days 1–28) with a control window (the remainder of the 240-day observation period, excluding any risk windows). We found 5,211 patients experienced FP within the risk window and 10,531 experienced FP within the control window. FP risk increased within 28 days postvaccination, primarily after first and second doses and was observed for both mRNA and viral vaccines. Clinicians should carefully assess the FP risk-benefit profile associated with the COVID-19 vaccines and monitor neurologic signs after vaccination.

Amid the COVID-19 pandemic, vaccines were widely distributed under emergency use authorizations worldwide (*1*,*2*). During the development phase of COVID-19 vaccines, although no severe safety concerns were evident in any of the pivotal clinical trials, an imbalance in facial palsy (FP) incidence after vaccination was observed in vaccinated persons compared with the general population (*3*–*6*). Although the exact etiology of FP remains elusive, infection, autoimmune mechanisms, or vaccination are considered potential contributors to its development (*7*,*8*). Because of its sudden and acute symptom onset, characterized by facial muscle paralysis, FP has been included in the priority list of adverse events of special interest generated by the Safety Platform for Emergency vACcines (SPEAC) (*9*).

Multiple studies on the association of FP after COVID-19 vaccination have been reported (*10*–*14*), but the results from those studies have been inconsistent and lack a clear consensus. The variability in study results may be attributed to several factors, including limited statistical power because of small study populations and heterogeneity among studies in terms of population, ethnicity, vaccine types, doses, observation periods, and statistical methods. Even though a systematic review and meta-analysis were conducted to address those concerns (*15*,*16*), reaching definitive conclusions was challenging because the limited definition of eligibility criteria in that analysis, such as types of studies or study participants, did not fully encompass all available evidence on FP.

Because of the controversial and inconclusive results of existing studies, previous evidence necessitates an in-depth body of evidence and a clear consensus on the safety of COVID-19 vaccines concerning FP. Using 2 large, linked databases in South Korea, we conducted a self-controlled case series analysis to address the inconsistent findings of previous studies and provide an updated overall assessment of the potential association between FP and COVID-19 vaccines.

## Methods

### Data Sources

This research was conducted as part of COVID-19 Vaccine Safety Research Committee (CoVaSC) in South Korea with the aim of providing evidence on the safety of COVID-19 vaccines for immunization. In South Korea, several types of COVID-19 vaccine were available during the study period: BNT162b2 (Pfizer-BioNTech, https://www.pfizer.com), mRNA-1273 (Moderna, https://www.modernatx.com), ChAdOx1 nCoV-19 (AstraZeneca, https://www.astrazeneca.com), Ad.26.COV2.S (Janssen, https://www.janssen.com), and NVX-CoV2373 (Novavax, https://www.novavax.com) (*17*). 

To obtain vaccine and adverse event data, we linked data from 2 large databases: the COVID-19 immunization registry (February 26, 2021–October 31, 2022) managed by the Korea Disease Control and Prevention Agency (KDCA) and healthcare claims data (January 1, 2002–October 31, 2022) provided by National Health Insurance Service (NHIS). During the COVID-19 pandemic in South Korea, KDCA and the government oversaw the distribution of vaccines and established the immunization registry covering the entire population. The registry included crucial information, such as age at vaccination, date of vaccination, type of vaccine administered, and dosing schedule of specific vaccines.

In accordance with the single-payer insurance provider system in South Korea, the NHIS covers the entire population of >50 million. The claims database of NHIS contains comprehensive healthcare utilization information on reimbursed patient visits, such as medical diagnoses, drug prescriptions, and medical screening data, which can be provided in an anonymized format. Diagnosis records are coded according to the International Classification of Disease 10th Revision (ICD-10), and drug prescriptions can be identified by national drug codes based on the Anatomic Therapeutic Chemical (ATC) classification of the World Health Organization.

This study followed the Strengthening the Reporting of Observational Studies in Epidemiology (STROBE) reporting guideline and was approved by the Public Institutional Review Board Designated by Ministry of Health and Welfare (approval no. P01-202203-01-005) and performed in accordance with the principles of the Declaration of Helsinki (World Medical Association, https://www.wma.net). The requirement of informed consent was waived because this study used anonymized administrative claims data.

### Study Population

**Figure 1:**
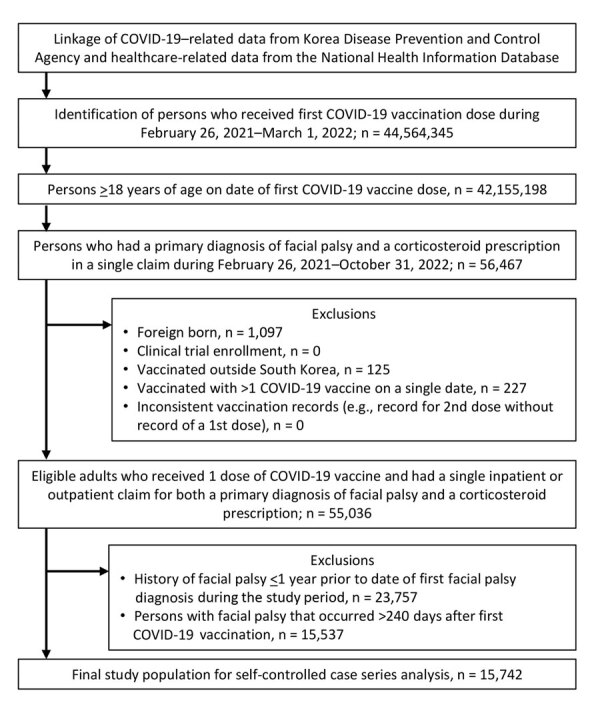
Flowchart of participant selection in study of risk for facial palsy after COVID-19 vaccination in South Korea, 2021–2022. The Korea Disease Control and Prevention Agency database has operated since 2020 and collects information on age, sex, type of vaccine and lot number, vaccinating healthcare provider, and date of vaccination for all COVID-19 vaccines. The National Health Information Database includes data collected by the National Health Insurance Service, which is the single-payer health insurance in South Korea, covering 97% of total population (≈50 million persons). The overall positive predictive value of the diagnoses recorded in the National Health Information Database is 82%.

We identified persons >18 years of age who received an initial COVID-19 vaccine dose during February 26, 2021–March 1, 2022. Among that population, we identified and included patients with a primary FP diagnosis accompanied by a prescription for corticosteroids during February 26, 2021–October 31, 2022. Because we adopted a self-controlled case series analysis (SCCS), we further included patients who received a COVID-19 vaccination and had incident FP diagnosed within a prespecified observation period. Exclusion criteria comprised foreign born residents, participants in clinical trials, persons vaccinated abroad, and persons deviating from vaccination guidelines (i.e., incomplete immunization information) to account for potential exposure misclassification. In addition, we excluded persons with a history of FP in the year preceding the observation period and those whose FP cases occurred after the end of observation period (Figure 1).

### SCCS Design

To investigate the risk for FP after COVID-19 vaccination, we used an SCCS design (*18*). SCCS uses within-person comparisons, which offers the advantage of minimizing the potential effects of time-invariant confounders that could be major limitations of the conventional cohort designs. We adopted the SCCS design because we recognized that selecting appropriate comparison groups would be challenging because of the high COVID-19 immunization rates in South Korea. We specified an observation period of 240 days after the first dose of any COVID-19 vaccine. We defined the risk window as days 1–28 after each dose of COVID-19 vaccination, with day 0 indicating the time of vaccination. We chose that timeframe as the risk window because persons are at a higher risk for FP occurrence during that period (Appendix Figure). We selected a 28-day risk window on the basis of previous studies (*10*,*19*) and observations from clinical trials of COVID-19 vaccines that indicated that neutralizing antibodies against SARS-CoV-2 peaked 28 days after vaccination (*20*,*21*). We defined the control windows as the periods outside the risk windows during the observation period.

### COVID-19 Vaccination

Since the implementation of a massive immunization campaign against COVID-19 in South Korea, KDCA has collected detailed information on immunization for each available vaccine. For the Ad26.COV2.S vaccine, a single dose was regarded as a complete primary series, whereas other vaccines required 2 doses for completion. We obtained immunization information on those COVID-19 vaccines and defined their receipt as exposures. To consider the potential effect of administering different types of vaccines, we categorized vaccinees as homologously vaccinated if they received the same type of COVID-19 vaccine throughout their dosing series and heterologously vaccinated otherwise. To assess the differential risk for FP occurrence on the basis of the biologic mechanism of action, we categorized specific vaccine types: BNT162b2 and mRNA-1273 as mRNA vaccines, ChAdOx1 nCoV-19 and Ad26.COV2.S as viral vector vaccines, and NVX-CoV2373 as a recombinant protein vaccine.

### Outcomes

The outcome of interest was FP, which we defined as a primary diagnosis of FP accompanied by a prescription for oral or parenteral corticosteroid on the same day. We included oral or parenteral corticosteroid prescription to enhance the outcome validity of FP because of the clinical context of administration to patients experiencing acute FP in South Korea. The CoVaSC clinical research committee reviewed and approved our case definition. Incident FP cases were identified by ICD-10 codes G51.0, G51.8, or G51.9, and corticosteroid prescription was identified by ATC code H02AB (Appendix Table 1). We observed all FP cases that occurred within the observation period in the eligible population and calculated the incidence rate ratio (IRR) by comparing the incidence rate of FP between the risk and control windows.

### Statistical Analysis

We summarized demographic characteristics according to the risk or control window, including age, sex, region of residence, health insurance type, and history of underlying conditions assessed <1 year before the first vaccine dose, including myocardial infarction, congestive heart failure, peripheral vascular disease, stroke, dementia, chronic pulmonary disease, rheumatic disease, peptic ulcer disease, mild liver disease, diabetes mellitus, diabetic complications, hemiplegia or paraplegia, renal disease, cancer, serious liver disease, solid or metastatic tumor, and HIV infection. Statistical analyses involved *t*-tests for continuous variables and χ^2^ tests for categorial variables. We considered p<0.05 statistically significant. 

We measured the number of events and person-years to estimate the incidence rate of FP in the risk and control windows. We used a conditional Poisson regression model to estimate IRRs and 95% CIs, comparing the FP incidence rates in the risk window with FP incidence in the control window. In secondary analyses, we explored vaccine-specific risks, considering doses (first, second, third, fourth, or first/second [a first dose of BNT162b2, mRNA-1273, ChAdOx1 nCoV-19, or Ad26.COV2.S and a second dose of BNT162b2, mRNA-1273, or ChAdOx1 nCoV-19]) and homologous or heterologous vaccination within the dose series.

We conducted subgroup analyses stratified by age groups (18–29, 30–39, 40–49, 50–59, 60–69, 70–79, >80 years of age), sex, type of insurance (health insurance or medical aid), region of residence (metropolitan or rural), Charlson Comorbidity Index score (<5 or >5), and history of underlying conditions. We applied a Benjamini-Hochberg adjustment to address the inflation of type I error resulting from multiple comparisons (*22*). 

To assess the robustness of our findings under various assumptions, we further conducted several sensitivity analyses. First, we repeated the main analysis by varying risk windows to 1–14 days or 1–42 days to assess the potential effects of different risk windows on the FP occurrence. Second, we excluded persons who died within 7 days after FP diagnosis to exclude susceptible persons. Third, because COVID-19 infection poses a potential risk factor for FP, as reported in previous studies (*23*–*25*), we excluded persons who had COVID-19 infection within 90 days before vaccination or who had COVID-19 infection before their FP diagnosis. Fourth, we restricted cases to inpatient or emergency department visits to minimize outcome misclassification due to the definition of outcome identification. Fifth, we further restricted cases to persons simultaneously prescribed corticosteroids and antiviral medications to consider various clinical aspect of managing FP in South Korea. Last, we restricted cases to Bell’s palsy diagnosis only (ICD-10 code G51.0) to account for the possibility of lower outcome validity of other FP diagnosis codes (ICD-10 codes G51.8 and G51.9). We used SAS Enterprise Guide version 8.3 (SAS Institute Inc., https://www.sas.com) for all statistical analyses.

## Results

A total of 44,564,345 persons in South Korea were administered 129,956,027 COVID-19 vaccine doses during February 26, 2021–March 1, 2022 (Figure 1). We identified 15,742 FP cases with corticosteroid prescriptions during the study period. Among those cases, 5,211 occurred within 1–28 days postvaccination, corresponding to 4.0 FP cases/1 million doses. Among the FP study population, the mean age at first COVID-19 vaccination was 53.1 (SD 15.9) years; 54.7% (n = 2,849) were male and 45.3% (n = 2,362) were female (Table 1).

**Table 1:** Baseline characteristics stratified by exposure windows in a study of risk for facial palsy after COVID-19 vaccination, South Korea, 2021–2022*

Baseline characteristics	Risk window, n = 5,211	Control window, n = 10,531	p value
Mean age, y (SD)	53.1 (15.9)	52.8 (16.0)	0.3147
Age group, y			0.1587
18–29	506 (9.7)	1,005 (9.5)	
30–39	597 (11.5)	1,310 (12.4)	
40–49	990 (19.0)	1,955 (18.6)	
50–59	1,204 (23.1)	2,472 (23.5)	
60–69	1,099 (21.1)	2,176 (20.7)	
70–79	599 (11.5)	1,113 (10.6)	
>80	216 (4.1)	500 (4.7)	
Sex			0.0417
M	2,849 (54.7)	5,938 (56.4)	
F	2,362 (45.3)	4,593 (43.6)	
Health insurance type			0.4262
National health insurance	5,045 (96.8)	10,170 (96.6)	
Medical aid	166 (3.2)	361 (3.4)	
Region of residence			0.7853
Metropolitan	3,485 (66.9)	7,020 (66.7)	
Rural	1,726 (33.1)	3,511 (33.3)	
Mean CCI (SD)	1.3 (1.8)	1.3 (1.7)	0.0540
CCI group			0.1488
CCI <5	4,866 (93.4)	9,896 (94.0)	
CCI <5	345 (6.6)	635 (6.0)	
Underlying conditions			
Myocardial infarction	41 (0.8)	115 (1.1)	0.0689
CHF	164 (3.1)	383 (3.6)	0.1144
Peripheral vascular disease	620 (11.9)	1,139 (10.8)	0.0424
Cerebrovascular disease	315 (6.0)	696 (6.6)	0.1742
Dementia	198 (3.8)	366 (3.5)	0.3031
CPD	676 (13.0)	1,408 (13.4)	0.4886
Rheumatic disease	137 (2.6)	301 (2.9)	0.4107
Peptic ulcer	848 (16.3)	1,634 (15.5)	0.2199
Mild liver disease	1,105 (21.2)	2,179 (20.7)	0.4553
Diabetes mellitus	1,190 (22.8)	2,236 (21.2)	0.0217
Diabetic complications	337 (6.5)	665 (6.3)	0.0289
Hemiplegia or paraplegia	20 (0.4)	56 (0.5)	0.2076
Renal disease	110 (2.1)	222 (2.1)	0.9906
Cancer	258 (5.0)	455 (4.3)	0.0734
Serious liver disease	15 (0.3)	21 (0.2)	0.2743
Solid metastatic tumor	22 (0.4)	32 (0.3)	0.2322
HIV infection	1 (0.0)	6 (0.1)	0.2900

*Values are no. (%) except as indicated. CCI, Charlson Comorbidity Index score; CHF, congestive heart failure; CPD, chronic pulmonary disease.

**Figure 2:**
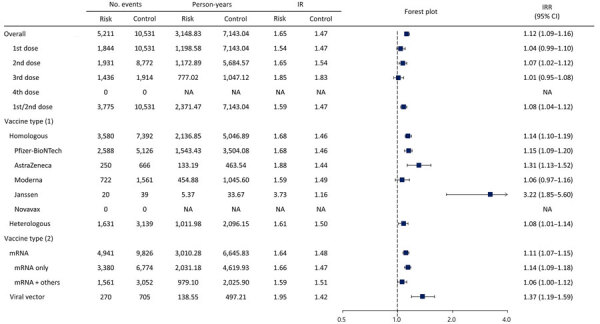
Forest plot of risk for facial palsy after COVID-19 vaccination in South Korea, 2021–2022. Plot assess facial palsy risk within 28 days of COVID-19 vaccination. Overall risk is shown, as is risk stratified by dose and vaccine type. Squares indicate IRRs; bars indicate 95% CIs. Vaccine types were BNT162b2 (Pfizer-BioNTech, https://www.pfizer.com), mRNA-1273 (Moderna, https://www.modernatx.com), ChAdOx1 nCoV-19 (AstraZeneca, https://www.astrazeneca.com), Ad.26.COV2.S (Janssen, https://www.janssen.com), and NVX-CoV2373 (Novavax, https://www.novavax.com). 1st/2nd dose indicates a first dose of BNT162b2, mRNA-1273, ChAdOx1 nCoV-19, or Ad26.COV2.S and a second dose of BNT162b2, mRNA-1273, or ChAdOx1 nCoV-19. IR, incidence rate; IRR, incidence rate ratio; NA, not applicable.

Our study showed FP risk increased within 1–28 days after any COVID-19 vaccine dose (IRR 1.12 [95% CI 1.09–1.16]). We observed increased FP risks (IRR 1.07 [95% CI 1.02–1.12]) with the second dose and combined first and second doses (IRR 1.08 [95% CI 1.04–1.12]) but identified no association for the third dose (IRR 1.01 [95% CI 0.95–1.08]). Regardless of whether persons received homologous or heterologous vaccination, we observed FP increased after vaccination; for homologous doses IRR was 1.14 (95% CI 1.10–1.19) and for heterologous doses IRR was 1.08 (95% CI 1.01–1.14). Furthermore, we found increased FP risks across vaccine types, in patients vaccinated with at least one mRNA vaccine IRR was 1.11 (95% CI 1.07–1.15) and in those vaccinated with viral vector vaccines only IRR was 1.37 (95% CI 1.19–1.59) (Figure 2; Appendix Table 2).

IRRs were generally consistent across age groups (Table 2; Appendix Table 3), and we identified elevated risks irrespective of sex. Among male persons, IRR was 1.08 (95% CI 1.03–1.13) and for female persons IRR was 1.18 (95% CI 1.12–1.24). After applying the Benjamini-Hochberg adjustment, those results generally remained consistent.

**Table 2:** Incidence risk and incidence risk ratios in a study of risk for facial palsy after COVID-19 vaccination, South Korea, 2021–2022*

Subgroup analyses	No. events			Person-years			IR		IRR (95% CI)
	Risk window	Control window		Risk window	Control window		Risk window	Control window	
Age group, y									
18–29	506	1,005		296.63	693.22		1.71	1.45	1.18 (1.06–1.31)†
30–39	597	1,310		345.53	907.82		1.73	1.44	1.20 (1.09–1.32)†
40–49	990	1,955		579.31	1,347.76		1.71	1.45	1.18 (1.09–1.27)†
50–59	1,204	2,472		760.05	1,647.95		1.58	1.50	1.06 (0.99–1.13)
60–69	1,099	2,176		683.97	1,453.30		1.61	1.50	1.07 (1.00–1.15)
70–79	599	1,113		351.39	760.24		1.70	1.46	1.16 (1.05–1.29)†
>80	216	500		131.96	332.74		1.64	1.50	1.09 (0.93–1.28)
Sex									
M	2,849	5,938		1,766.69	3,981.28		1.61	1.49	1.08 (1.03–1.13)†
F	2,362	4,593		1,382.14	3,161.77		1.71	1.45	1.18 (1.12–1.24)†
Health insurance type									
National health insurance	5,045	10,170		3,043.69	6,908.08		1.66	1.47	1.13 (1.09–1.16)†
Medical aid	166	361		105.15	234.96		1.58	1.54	1.03 (0.86–1.23)
Region of residence									
Metropolitan	3,485	7,020		2,099.87	4,770.18		1.66	1.47	1.13 (1.08–1.17)†
Rural	1,726	3,511		1,048.96	2,372.86		1.65	1.48	1.11 (1.05–1.18)†
Charlson Comorbidity Index score									
<5	4,866	9,896		2,948.41	6,705.30		1.65	1.48	1.12 (1.08–1.16)†
>5	345	635		200.43	437.75		1.72	1.45	1.19 (1.04–1.35)†

*Date of vaccination considered day 0, risk window considered days 1–28 postvaccination, and control window considered days 29–240 postvaccination. IR, incidence rate; IRR, incidence rate ratio. †These results remained significant (p<0.05) after applying the Benjamini-Hochberg adjustment to account for multiple comparisons (*22*).

**Figure 3:**
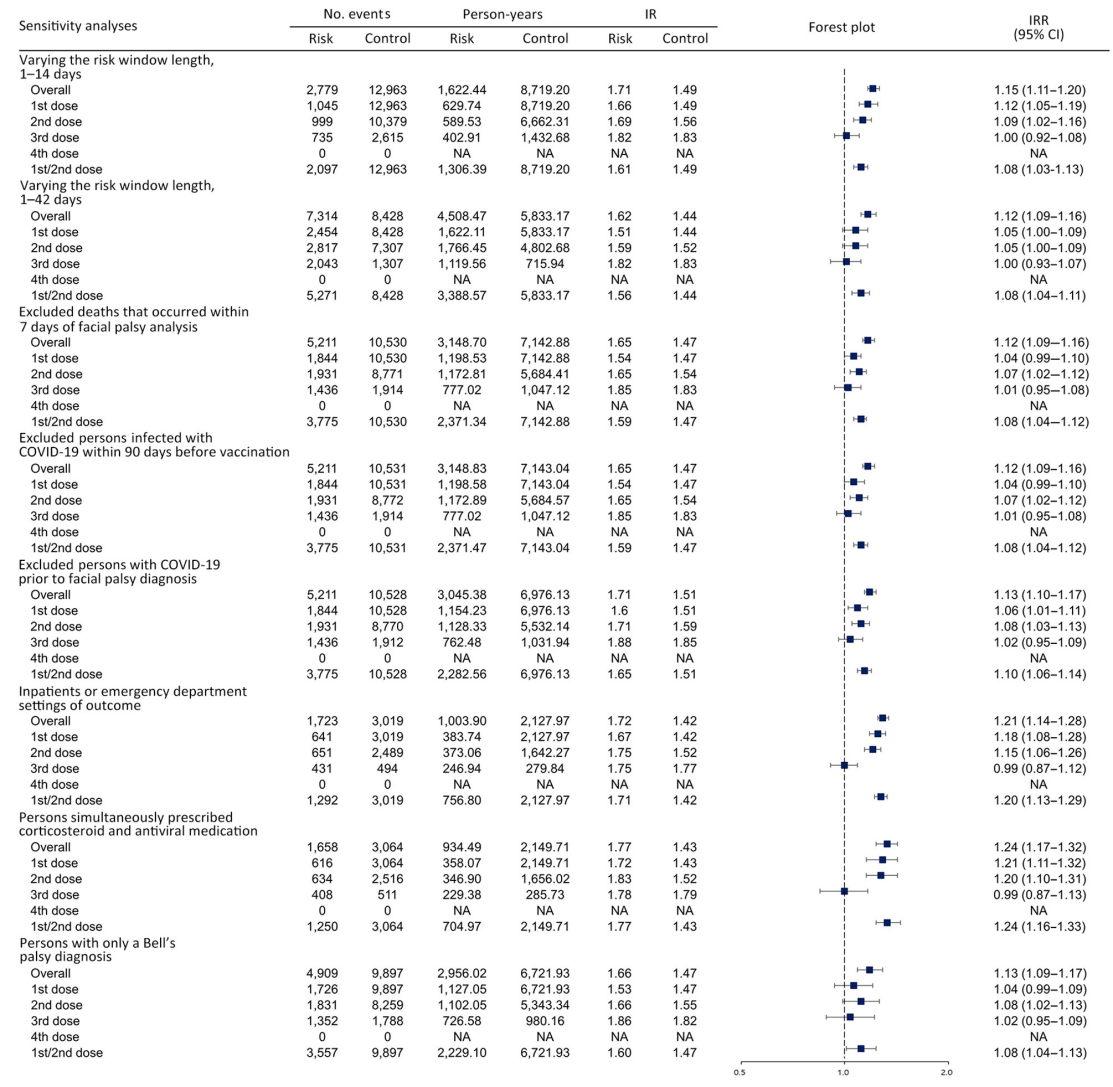
Forest plot of sensitivity analyses of risk for facial palsy after COVID-19 vaccination in South Korea, 2021–2022. Overall risk is shown, as is risk stratified by adverse events of interest. Squares indicate IRRs; bars indicate 95% CIs. 1st/2nd dose indicates a first dose of BNT162b2, mRNA-1273, ChAdOx1 nCoV-19, or Ad26.COV2.S and a second dose of BNT162b2, mRNA-1273, or ChAdOx1 nCoV-19. IR, incidence rate; IRR, incidence rate ratio; NA, not applicable.

Sensitivity analyses demonstrated the robustness of the main results. The results remained consistent across both shorter and longer risk windows. IRR was 1.15 (95% CI 1.11–1.20) for the 1–14-day window and 1.12 (95% CI 1.09–1.16) for the 1–42-day window. Excluding persons who died within 7 days after FP diagnosis showed the FP risk was comparable to the main findings (IRR 1.12 [95% CI 1.09–1.16]). Moreover, we noted increased risks for FP regardless of COVID-19 infection, and those risks increased when we excluded cases of COVID-19 infection within 90 days before vaccination (IRR 1.12 [95% CI 1.09–1.16]) and COVID-19 cases before FP diagnosis (IRR 1.13 [95% CI 1.10–1.17]). Elevated risks of FP were shown when we restricted cases to inpatient or emergency department visits (IRR 1.21 [95% CI 1.14–1.28), to persons simultaneously prescribed corticosteroids and antiviral medication (IRR 1.24 [95% CI 1.17–1.32]), and to Bell’s palsy diagnosis (IRR 1.13 [95% CI 1.09–1.17]) (Figure 3).

## Discussion

Using 2 large, linked databases from the national COVID-19 immunization registry and NHIS claims data, we identified a positive association between COVID-19 vaccination and FP in the population of South Korea. The overall transient risk for postvaccination FP was primarily determined by the events that occurred within 28 days after the first and second doses of COVID-19 vaccines. We observed increased FP risks across all vaccine types, among homologous and heterologous vaccinees, and for mRNA and viral vaccines.

Our findings contribute to the evidence of a positive association between FP and COVID-19 vaccination, aligning with other studies. In a study of 2.6 million patients in Israel vaccinated with BNT162b2 during December 20, 2020–April 30, 2021, the standardized FP IRR at 21 days after the first dose was 1.36 (95% CI 1.14–1.61) compared with the period before the COVID-19 pandemic (*11*). Another SCCS analysis in the United Kingdom showed a positive association with FP for ChAdOx1 nCoV-19 vaccine during the 15–21 days after vaccination (IRR 1.29 [95% CI 1.08–1.56]) (*14*). Furthermore, our study aligns with a population-based study conducted in Hong Kong, China, which reported an overall increased risk for Bell’s palsy after the first and second BNT162b2 vaccinations (adjusted odds ratio 1.54 [95% CI 1.12–2.12]) (*10*). Specifically, that study reported a substantially increased risk for Bell’s palsy within the first 14 days after the second dose in both nested case–control (adjusted odds ratio 2.33 [95% CI 1.41–3.82]) and SCCS (IRR 2.44 [95% CI 1.32–4.50]) analyses (*10*). Those findings are consistent with our findings, which also showed an increased risk for FP after the second COVID-19 vaccine dose.

Although the exact biological mechanism for development of FP after vaccination is unknown, plausible links between FP and both mRNA and viral vector COVID-19 vaccines have been proposed. First, mRNA vaccines use lipid nanoparticles to encapsulate SARS-CoV-2 spike antigen (*26*). When the mRNA lipid nanoparticles are recognized as foreign materials, the innate immune system is induced, stimulating production of type I interferons (*27*–*29*). As the immune response acts against myelin basic proteins, proinflammatory cytokines are profoundly released, damaging the myelin sheath and thereby attenuating peripheral tolerance (*30*–*32*). That proposed mechanism is supported by prior studies where Bell’s palsy occurred in patients undergoing interferon therapy (*33*,*34*). 

Similar to results for previous studies (*10*,*35*), our study revealed increased FP risks in persons homologously vaccinated with mRNA vaccines, especially for BNT162b2 (IRR 1.15 [95% CI 1.09–1.12]) and in those with at least a single dose of mRNA vaccine (IRR 1.11 [95% CI 1.07–1.15]). In addition, viral vector vaccines may trigger production of antibodies against virus proteins. Because of molecular mimicry between viral and peripheral nerve antigens, those antibodies can react with myelin antigens, causing demyelination. In addition, bystander activation of autoreactive T cells by viral vector vaccines can also provoke autoimmune phenomena (*36*,*37*). In line with a previous study that showed high T-cell responses after ChAdOx1 nCoV-19 vaccination (*38*), our study revealed elevated risks FP among patients who received homologous dosing of viral vector vaccines: IRR 1.31 (95% CI 1.13–5.52) for ChAdOx1 nCoV-19 and IRR 3.22 (95% CI 1.85–5.60) for Ad26.COV2.S vaccines. Moreover, recipients of viral vector vaccines had a much higher risk for FP (IRR 1.37 [95% CI 1.19–1.59]). However, that interpretation should be approached with caution because of the small number of FP cases, particularly with Ad26.COV2.S vaccines.

Recent studies have suggested that COVID-19 infection itself could also be a risk factor for FP onset (*23*) because it may lead to nerve compressions resulting from inflammation in response to viral infections (*39*). In South Korea, the annual incidence of Bell’s palsy increased from 23.0 to 30.8 cases/100,000 persons from 2008 to 2018 (*40*) and reached 32.5 cases/100,000 persons during 2021–2022 (*24*), suggesting an increasing trend during the COVID-19 pandemic. In addition, a retrospective cohort study in South Korea indicated that COVID-19 infection is associated with a higher risk for Bell’s palsy for both COVID-19 vaccine recipients (IRR 1.20 [95% CI 1.15–1.25]) and nonrecipients (IRR 1.84 [95% CI1.59–2.12]) (p<0.001) (*24*). In our SCCS study, a design widely used for vaccine safety evaluation, postvaccination FP risk was identified despite the previously recognized risk for FP after COVID-19 infection. The postvaccination risk is further supported by the consistent increase in FP risk observed in our sensitivity analysis, in which we excluded persons infected with COVID-19 from the study cohort.

By using a large, linked database in South Korea that covered >44 million persons vaccinated with >130 million vaccine doses, our study revealed an increased risk for FP after COVID-19 vaccination, providing supportive real-world evidence on postvaccination FP. We could address the inconsistencies observed in previous studies resulting from various limitations, including limited statistical power resulting from a small number of FP cases (*13*), and heterogeneity in vaccine types and doses studied for each analysis.

The first limitation of our study is the possible misclassification of FP cases because we relied on ICD-10 codes and could not apply the Brighton Collaboration’s definition for FP because of the lack of laboratory data in our database (*41*). Nevertheless, we defined our case definition to include only FP patients with prescriptions for corticosteroids, and we applied several other definitions of FP in sensitivity analyses to assess the robustness of our main results. The second limitation is that the actual timing of FP occurrence and diagnosis recorded might differ, potentially leading cases to be included in control window. Nevertheless, our sensitivity analyses by varying the length of risk windows showed comparable findings. Furthermore, even though we conducted sensitivity analyses to adjust for the effects of COVID-19 infection, residual confounding may remain among patients who did not undergo a COVID-19 testing and were later received an FP diagnosis or COVID-19 vaccination.

In conclusion, our study revealed a transient risk for FP after any dose of COVID-19 vaccine, irrespective of homologous and heterologous dosing or vaccine type. However, of note, although the risk for FP appears elevated, the absolute number of FP cases was small, and risk for FP should not discourage patients from receiving COVID-19 vaccinations. Because FP is generally mild and manageable, physicians should monitor neurologic signs after COVID-19 vaccination and provide patients with a comprehensive evaluation of the risk–benefit profile associated with COVID-19 vaccines.

Appendix Additional information on risk for facial palsy after COVID-19 vaccination, South Korea, 2021–2022.
