# Supplementary material for: Risk for Facial Palsy after COVID-19 Vaccination, South Korea, 2021–2022
Source: Emerg Infect Dis. 2024 Nov;30(11):2313–22. doi: 10.3201/eid3011.240610 (PMC11521192; doi:10.3201/eid3011.240610)
Supplement: Appendix — Additional information on risk for facial palsy after COVID-19 vaccination, South Korea, 2021–2022. [file 24-0610-Techapp-s1.pdf]

Article DOI: https://doi.org/10.3201/eid3011.240610

EID cannot ensure accessibility for supplementary materials supplied by authors. Readers who have difficulty accessing supplementary content should contact the authors for assistance.

Risk for Facial Palsy after COVID-19 Vaccination, South Korea, 2021–2022

Appendix Table 1. Facial palsy case definition based on diagnostic and prescription codes*

Codes	Diagnoses and prescriptions
ICD-10 codes	
G51.0	Bell's palsy
G51.8	Other disorders of facial nerve
G51.9	Disorders of facial nerve, unspecified
ATC code	Drug prescription
H02AB01	Betamethasone
H02AB02	Dexamethasone
H02AB04	Methylprednisolone
H02AB06	Prednisolone
H02AB08	Triamcinolone
H02AB09	Hydrocortisone
H02AB13	Deflazacort

*ATC, Anatomic Therapeutic Chemical; ICD-10, International Classification of Diseases, 10th Revision.

Appendix Table 2. Number of facial palsy cases by COVID-19 vaccine dose and exposure window

Vaccine doses	Risk window, n = 5,211	Control window, n = 10,531	p value
First dose type			0.0075
Pfizer-BioNTech	2,626	5,158	
Novavax	0	0	
Moderna	789	1,636	
Janssen	134	373	
AstraZeneca	1,662	3,364	
Second dose type			<0.0001
Pfizer-BioNTech	2,692	5,614	
Novavax	11	4	
Moderna	685	1,568	
AstraZeneca	1,362	2,793	
Not vaccinated	461	552	
Third dose type			<0.0001
Pfizer-BioNTech	2,276	4,309	
Novavax	22	16	
Moderna	1,315	2,605	
Janssen	2	5	
Not vaccinated	1,596	3,596	
Fourth dose type			0.6183
Pfizer-BioNTech	4	4	
Not vaccinated	5,207	10,527	

Appendix Table 3. Risk of facial palsy within 28 d of COVID-19 vaccination, stratified by age, sex, insurance type, region and underlying conditions*

	No. events		Person-years		IR		
Subgroup analyses	Risk	Control	Risk	Control	Risk	Control	IRR (95% CI)
Age group, y							
18–29	506	1,005	296.63	693.22	1.71	1.45	1.18 (1.06–1.31)†
30–39	597	1,310	345.53	907.82	1.73	1.44	1.20 (1.09–1.32)†
40–49	990	1,955	579.31	1,347.76	1.71	1.45	1.18 (1.09–1.27)†
50–59	1,204	2,472	760.05	1,647.95	1.58	1.50	1.06 (0.99–1.13)
60–69	1,099	2,176	683.97	1,453.30	1.61	1.50	1.07 (1.00–1.15)
70–79	599	1,113	351.39	760.24	1.70	1.46	1.16 (1.05–1.29)†
≥80	216	500	131.96	332.74	1.64	1.50	1.09 (0.93–1.28)
Sex							
M	2,849	5,938	1,766.69	3,981.28	1.61	1.49	1.08 (1.03–1.13)†
F	2,362	4,593	1,382.14	3,161.77	1.71	1.45	1.18 (1.12–1.24)†
Health insurance type							
National health insurance	5,045	10,170	3,043.69	6,908.08	1.66	1.47	1.13 (1.09–1.16)†
Medical aid	166	361	105.15	234.96	1.58	1.54	1.03 (0.86–1.23)
Region of residence							
Metropolitan	3,485	7,020	2,099.87	4,770.18	1.66	1.47	1.13 (1.08–1.17)†
Rural	1,726	3,511	1,048.96	2,372.86	1.65	1.48	1.11 (1.05–1.18)†
Charlson comorbidity index							
<5	4,866	9,896	2,948.41	6,705.30	1.65	1.48	1.12 (1.08–1.16)†
≥5	345	635	200.43	437.75	1.72	1.45	1.19 (1.04–1.35)†
History of myocardial infarction							
N	5,170	10,416	3,116.56	7,073.48	1.66	1.47	1.13 (1.09–1.17)†
Y	41	115	32.28	69.57	1.27	1.65	0.77 (0.54–1.10)
History of congestive heart failure							
N	5,047	10,148	3,037.79	6,896.91	1.66	1.47	1.13 (1.09–1.17)†
Y	164	383	111.04	246.13	1.48	1.56	0.95 (0.79–1.14)
History of peripheral vascular disease							
N	4,591	9,392	2,790.82	6,353.69	1.65	1.48	1.11 (1.07–1.15)†
Y	620	1,139	358.01	789.36	1.73	1.44	1.20 (1.09–1.32)†
History of stroke							
N	4,896	9,835	2,942.02	6,692.42	1.66	1.47	1.13 (1.09–1.17)†
Y	315	696	206.81	450.62	1.52	1.54	0.99 (0.86–1.13)
History of dementia							
N	5,013	10,165	3,038.40	6,886.86	1.65	1.48	1.12 (1.08–1.16)†
Y	198	366	110.43	256.18	1.79	1.43	1.26 (1.06–1.49)†
History of chronic pulmonary disease							
N	4,535	9,123	2,727.53	6,209.38	1.66	1.47	1.13 (1.09–1.17)†
Y	676	1,408	421.30	933.66	1.60	1.51	1.06 (0.97–1.17)
History of rheumatic disease							
N	5,074	10,230	3,060.15	6,944.08	1.66	1.47	1.13 (1.09–1.16)†
Y	137	301	88.68	198.96	1.54	1.51	1.02 (0.83–1.25)
History of peptic ulcer disease							
N	4,363	8,897	2,637.37	6,021.89	1.65	1.48	1.12 (1.08–1.16)†
Y	848	1,634	511.46	1,121.15	1.66	1.46	1.14 (1.05–1.24)†
History of mild liver disease							
N	4,106	8,352	2,474.94	5,672.21	1.66	1.47	1.13 (1.09–1.17)†
Y	1,105	2,179	673.89	1,470.84	1.64	1.48	1.11 (1.03–1.19)†
History of diabetes mellitus							
N	4,021	8,295	2,441.09	5,615.50	1.65	1.48	1.12 (1.07–1.16)†
Y	1,190	2,236	707.74	1,527.55	1.68	1.46	1.15 (1.07–1.23)†
History of diabetic complications							
N	4,834	9,866	2,936.03	6,678.14	1.65	1.48	1.11 (1.08–1.15)†
Y	377	665	212.80	464.90	1.77	1.43	1.24 (1.09–1.41)†
History of hemiplegia/paraplegia							
N	5,191	10,475	3,133.70	7,108.81	1.66	1.47	1.12 (1.09–1.16)†
Y	20	56	15.13	34.23	1.32	1.64	0.81 (0.48–1.36)
History of renal disease							
N	5,101	10,309	3,081.30	6,993.62	1.66	1.47	1.12 (1.09–1.16)†
Y	110	222	67.53	149.43	1.63	1.49	1.10 (0.87–1.38)
History of cancer							
N	4,953	10,076	3,002.51	6,823.69	1.65	1.48	1.12 (1.08–1.16)†
Y	258	455	146.32	319.35	1.76	1.42	1.24 (1.06–1.44)†

	No. events		Person-years		IR		
Subgroup analyses	Risk	Control	Risk	Control	Risk	Control	IRR (95% CI)
History of serious liver disease							
N	5,196	10,510	3,141.33	7,127.18	1.65	1.47	1.12 (1.08–1.16)†
Y	15	21	7.50	15.86	2.00	1.32	1.51 (0.79–2.90)
History of solid/metastatic tumor							
N	5,189	10,499	3,138.19	7,118.44	1.65	1.47	1.12 (1.08–1.16)†
Y	22	32	10.64	24.61	2.07	1.30	1.59 (0.90–2.80)
History of HIV infection							
N	5,210	10,525	3,146.79	7,138.98	1.66	1.47	1.12 (1.09–1.16)†
Y	1	6	2.05	4.07	0.49	1.47	0.33 (0.04–2.66)

*IR, incidence rate; IRR, incidence rate ratio.
†These results remained significant after applying the Benjamini-Hochberg adjustment to account for multiple comparisons.

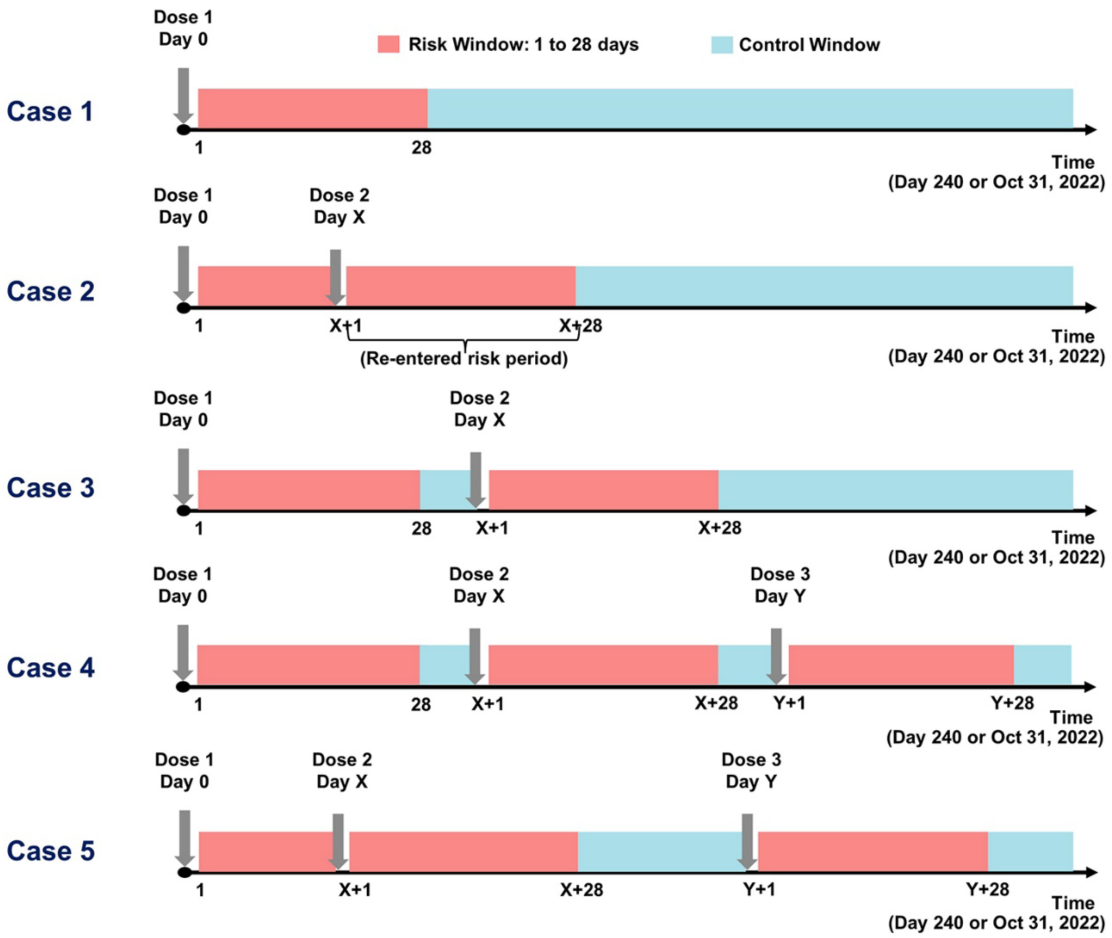

Appendix Figure. Self-controlled case series design assessing the risk of new-onset facial palsy following COVID-19 vaccinations is illustrated.
